# Optical Twist Induced by Plasmonic Resonance

**DOI:** 10.1038/srep27927

**Published:** 2016-06-13

**Authors:** Jun Chen, Neng Wang, Liyong Cui, Xiao Li, Zhifang Lin, Jack Ng

**Affiliations:** 1 Department of Physics, Hong Kong Baptist University, Hong Kong, China; 2 Institute of Theoretical Physics and Collaborative Innovation Center of Extreme Optics, Shanxi University, Shanxi, China; 3 State Key Laboratory of Surface Physics, Key Laboratory of Micro and Nano Photonic Structures (MOE), Collaborative Innovation Center of Advanced Microstructures, and Department of Physics, Fudan University, Shanghai, China; 4 Institute of Computational and Theoretical Studies, Hong Kong Baptist University, Kowloon Tong, Hong Kong, China

## Abstract

Harvesting light for optical torque is of significant importance, owing to its ability to rotate nano- or micro-objects. Nevertheless, applying a strong optical torque remains a challenging task: angular momentum must conserve but light is limited. A simple argument shows the tendency for two objects with strong mutual scattering or light exchange to exhibit a conspicuously enhanced optical torque without large extinction or absorption cross section. The torque on each object is almost equal but opposite, which we called optical twist. The effect is quite significant for plasmonic particle cluster, but can also be observed in structures with other morphologies. Such approach exhibits an unprecedentedly large torque to light extinction or absorption ratio, enabling limited light to exert a relatively large torque without severe heating. Our work contributes to the understanding of optical torque and introduces a novel way to manipulate the internal degrees of freedom of a structured particle cluster.

Strong optical torques^1^,^2^,^3^,^4^,^5^,^6^,^7^,^8^,^9^,^10^,^11^,^12^ typically involve strong light extinction and absorption. Such approach is ultimately limited by the availability of light and heating. Here, we report a large optical torque acting on two nearby particle clusters, mimicked by configurations in Fig. 1. To ensure numerical convergence, each particle cluster is made up of identical spheres lying on a plane perpendicular to the *z*-axis. This model of particle clusters serves as a proof of concept demonstration: one can achieve a strong torque at relatively low extinction and absorption rate. Strong light exchange among the particle clusters results in nearly equal and opposite torques (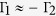). Hereafter, we shall refer the relative torque, defined as 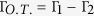, as the optical twist.

Upon illumination by a beam with positive angular momentum, the lower particle cluster is likely to be rotated in the positive direction^12^. Then by angular momentum conservation, angular momentum in the scattered light will be reduced (i.e. if the object gains positive angular momentum, then the field will gain negative angular momentum, owing to angular momentum conservation), which rotates the upper particle cluster in the negative sense. Similarly, the angular momentum of light scattered from the upper particle cluster increases, which enhances the positive rotation of the lower particle cluster. Thus, in this multiple scattering process, the lower and upper particle clusters will exhibit enhanced positive and negative torques, respectively, allowing a large optical twist.

Significant optical twists can be observed in high dielectric or metallic particle cluster. Such twists, because the torques on each particle cluster are equal and opposite, are significantly stronger than the total torque (Γ_*total*_ = Γ_1_ + Γ_2_) acting on the double particle clusters or the torque on a similar single particle cluster. More importantly, the torque per unit light energy extinction rate (Γ_*z*_/*W*_ext_) and absorption rate (Γ_*z*_/*W*_abs_) are much greater than those of the single particle cluster, allowing limited light to apply a relatively large torque without severe heating. Last but not least, compared with direct light illumination, a metallic structure is able to shape the light beam to exert torque on a dielectric object in a much more effective manner. This will add more possibilities for optical manipulation.

## Results

### Optical twist for metallic materials

The optical torque and twist for structures made up of silver spheres are plotted in Fig. 2. The structure serves as the prototype for more realistic structures. The exact value of the torque can be different between our structure and the realistic ones, the aim here is to demonstrate the possibility of achieving large torque without increasing the laser power (or energy extinction and absorption rates).

The effect of optical twist is evident for the silver structure (see Fig. 2(c)), where the dielectric constant of silver is modeled by the Drude model:


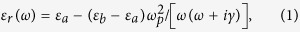


where *ε*_*a*_ = 5.45, *ε*_*b*_ = 6.18, *ω*_*p*_ = 1.72 × 10^16^ *rad*/*s*, *γ* = *ν*_*F*_/*l*, *ν*_*F*_ = 1.38 × 10^6^ *m*/*s*, and *l* = 52 *nm*^13^,^14^.

We remark that the resonance torque can drive the two particle clusters to rotate in the same or opposite direction, marked by red and black arrows on Fig. 2(c), respectively. This indicates the possibility of manipulating the internal degree of freedom at will by using light of different frequency.

Moreover, the optical twist or torque per unit energy extinction rate and per unit absorption rate for both single- and double-particle-cluster structures are plotted in Fig. 2(e,f), respectively. A large Γ_*O.T*._/*W*_ext_ allows one to exert a relatively large torque without extracting a large amount of light from the incident beam. Whereas a large Γ_*O.T*._/*W*_abs_ allows a strong torque without severe heating. Denoting the optical torque of the single particle cluster by Γ_single_, for silver particle clusters in Fig. 2(e), Γ_*O.T*._/*W*_ext_ is sharply enhanced compare to Γ_single_/*W*_ext_, indicating the existence of strong light exchange associated with resonance at hybridized plasmonic modes^14^. Figure 2(f) shows that Γ_*O.T*._/*W*_abs_ is also significantly enhanced for nearly all frequencies. This indicates that large torque without strong absorption is possible with our approach. These properties clearly illustrate the advantages of our approach of torque extraction – with its small light extinction and absorption, our approach can achieve something that a single particle cluster cannot.

### The role of particle-particle gap to optical twist

The role of gap to the torque are depicted in Fig. 3(b-d) with the structure shown in Fig. 3(a). In Fig. 3(b), wavelength is arbitrarily fixed at 482 nm (f = 623 THz). As the separation between adjacent spheres (denoted by “*D*”) is varied, resonances are triggered at some particular *D*. The strength of the optical twist is generally significantly stronger than that of the total torque acting on the double particle clusters. Figure 3(c) shows that the effects are similar for different *D* ranging from a 0.8 nm gap (60 + 0.8 = 60.8 nm) to a 10 nm gap. For a *D* comparable to wavelength, the effect should diminish and disappear. Optical twist is also found to exist in a wide range of intra-cluster gap (i.e., the gap between the spheres of the same layer, denoted by “Δ”). Figure 3(d) plotted the optical twist vesus Δ (ranged from 0.8 to 30 nm), while keeping the gap between the two particle clusters fixed. Clearly, strong optical twist exists in all cases shown, although they differ in the detailed profiles and resonance positions.

### Effect of mis-alignment in the two particle clusters

The structure in Fig. 2 consists of a stack of two particle clusters. Consider the structure obtained by manually rotating the upper particle cluster, as shown in Fig. 4(a). The optical torque is plotted in Fig. 4(b-d). One can see that the toque is actually small for rotation angle close to zero. At those angles, owing to symmetry, the resonance forces is basically parallel to the line joining the sphere centers (z-axis), which cannot induce a strong *z*-component torque, due to the short moment arm. The torque is not exactly zero at zero rotation angles because the incident light is circularly polarized. As the upper layer is rotated, the hybridization forces^14^, due to the bonding and antibonding coupling of the nanospheres, are no longer parallel to moment arm and can now induce a torque more effectively. Accordingly, the torque in Fig. 4(d) increases sharply. However, with further manual rotation, the larger separation makes the short range resonance diminishes rapidly and the torque decreases.

It was shown in ref. 14 that at the assumed intensity, the attractive bonding force is on the order of 0.5 *pNμm*, which is approximately 50 times stronger than the anti-bonding force (~0.01 *pNμm*). Moreover, the already weak anti-bonding force will be further reduced by the absorption of circularly polarized incident wave, which induces an absorption torque of approximately 0.005 *pNμm* per cluster. While this is small compared to the bonding force, but it may partially or fully cancel the torque from the anti-bonding mode. Accordingly, the anti-bonding force can hardly be seen on the scale of Fig. 4.

### Optical twist associated with different particle morphologies and beam profiles

Optical twist could be observed in a wide class of double layer configurations. In addition to the structure in Fig. 2, more examples are given in Fig. 5. For the four silver spheres structure in Fig. 5(a), the magnitude of the twist is limited by its small structural size. Evidently, the larger structures shown in Fig. 5(c,e) exhibit significantly stronger twists. This is because a larger size attracts more light and it also enhances multiple scattering. The optical twist is observed for an irregular structure plotted in Fig. 5(e,f). As a last example, the optical twist for a system illuminated by a *l* = 1 Laguerre Gaussian beam carrying orbital angular momentum is also plotted in Fig. 5(g,h), which shows similar optical twist.

### Shaping beam to rotate dielectric objects

Due to the low dielectric contrast, it is difficult to apply a large optical torque on a dielectric structure, as shown in Fig. 6 (the thick black line). Figure 6 shows the possibility of using a metallic structure as an antenna to shape the incident beam to rotate a dielectric object. The torque acting on the dielectric (glass) with metal below it (the thick purple line) is two orders of magnitude stronger than that without the metal (the thick black line). On the contrary, the torque on metal does not change appreciably. More surprisingly, the glass structure can also be rotated in the negative sense (relative to the incident angular momentum). We are aware of the vigorous development of plasmonic trapping^15^,^16^,^17^,^18^,^19^,^20^,^21^,^22^,^23^,^24^,^25^,^26^,^27^,^28^,^29^, and our work here can be considered as its extension, which provide additional rotational degrees of freedom in manipulation.

## Discussion

We investigated the optical twist in two-particle-cluster structures. One of its advantages is the ability to recycle light by resonance light exchange between the two or more particle clusters, which induces a significant enhancement in the optical torque to light extinction ratio. While one can also create strong optical torque by plasmonic resonance^8^, it inevitably associated with high absorption, which is the origin of many undesirable effects. The torque per unit energy absorption rate in the double-particle-cluster structure is much larger than that of the single particle cluster. This eliminates many undesirable effect such as heating.

Albeit we focus on silver nanoparticle cluster, such effect can also be found in other nanostructures where internal multiple scattering is strong. The high dielectric cluster is another example. Figure 7(a) shows that, multiple scattering is weak for polystyrene particle cluster (*ε*_*r*_ = 2.4649), thus the twist is also weak, and Γ_*O.T*._/*W*_ext_ is comparable to Γ_single_/*W*_ext_ (see Fig. 7(c)). The twist is much more significant for high dielectric (*ε*_*r*_ = 12.25, see Fig. 7(b)), where the high-*Q* Mie resonance traps the light for a relatively long time, thus enhances the intensity and light matter interaction. Moreover, Γ_*O.T*._/*W*_ext_ is also much greater than Γ_single_/*W*_ext_ (see Fig. 7(d)). We note that the radius of spheres in Fig. 2(c,d) is almost seven times smaller than those used in Fig. 7(a,b).

While our approach can achieve something a single particle cannot, it is a consequence of physical law such as angular momentum conservation. In fact, angular momentum conservation has been taken into account fully and automatically by our Mie scattering theory and Maxwell stress tensor formalism.

For a single particle illuminated by light, the azimuthal channel of the scattered wave must obey the azimuthal selection rule:


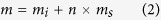


where *m*_*i*_ is the azimuthal index for the incident wave, *m*_*s*_ is the degree of discrete rotational symmetry of the particle cluster under light illumination, and *n* = 0, ±1, ±2 and so on. The particle will then experience a recoil torque of −*n* × *m*_*s*_ due to angular momentum conservation. While it is true that the larger the *n*, the larger the recoil torque, scattering a photon into a large *n* channel is highly unlikely, due to the little overlap between a large *n* scattered field and the relatively small particle. This can be seen more clearly from a classical point of view: **L** = **r** × **p** and a free space photon has a fixed |**p**| = *ħk* at a particular frequency. Consequently, a photon with large angular momentum cannot have a small **r**, implying that it cannot reach a small particle in the origin. The situation for a pair of nearby particles is completely different. Roughly speaking, the evanescent wave from the lower particle cluster can have a |**p**| ≫ *ħk* that can interact with the upper particle cluster, and vice versa. This would allow a larger recoil torque, and thus a larger optical twist. We stress that it is the multiple scattering of light between the two layers of particles that produce the enhanced optical twist.

The strong torque for the high dielectrics and metals can also be understood from the viewpoint of hybridization^14^,^30^,^31^,^32^,^33^ of the optical modes of individual component in the nanostructure, mimicked here by cluster of spheres. In the ideal case, when the two layers are brought to the vicinity of each other, each of their optical modes splits into an attractive bonding one and a repulsive anti-bonding mode^14^,^34^,^35^,^36^. A realistic situation is more complicated. Multiple optical modes can be involved in the hybridization process, resulting in complicated interaction. In any case, according to the following equation^37^:


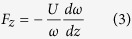


which is valid for a closed system and approximately valid for a resonance system, the modes decrease in frequency exerts an attraction between the two particles, whereas those increase in frequency exerts a repulsive force^37^. These attractive and repulsive forces correspond to strong optical torque in opposite directions.

The bi-layer cluster structure we considered may not be an easily experimentally accessible system. However, as we demonstrated above, the existence of optical twist does not depend critically on inter and intra layer gaps. Accordingly, optical twist should be realizable in a wide range of plasmonic structure, such as a pair of closely spaced structured thin film.

## Methods

**Figure 1:**
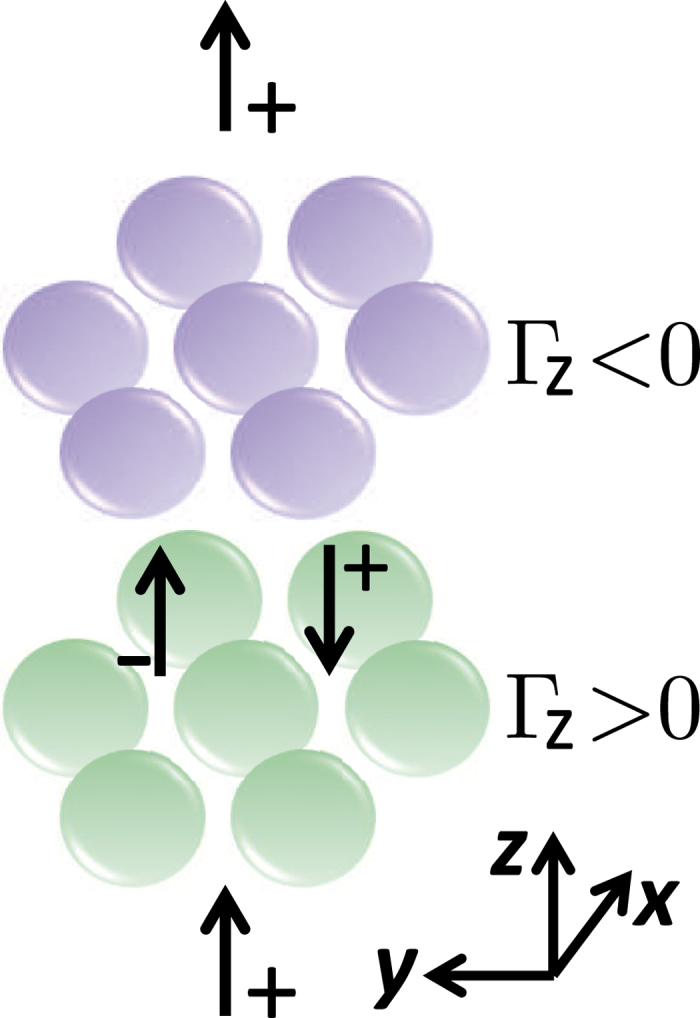
The double-particle-cluster configuration. The arrows denote the propagation direction of the light, with + and − denoting positive and negative spin angular momentum, respectively. For structures with strong mutual scattering or light exchange, the torques acting on the bottom and top particle clusters are likely to be equal and opposite as depicted in the figure and explained in the main text.

**Figure 2:**
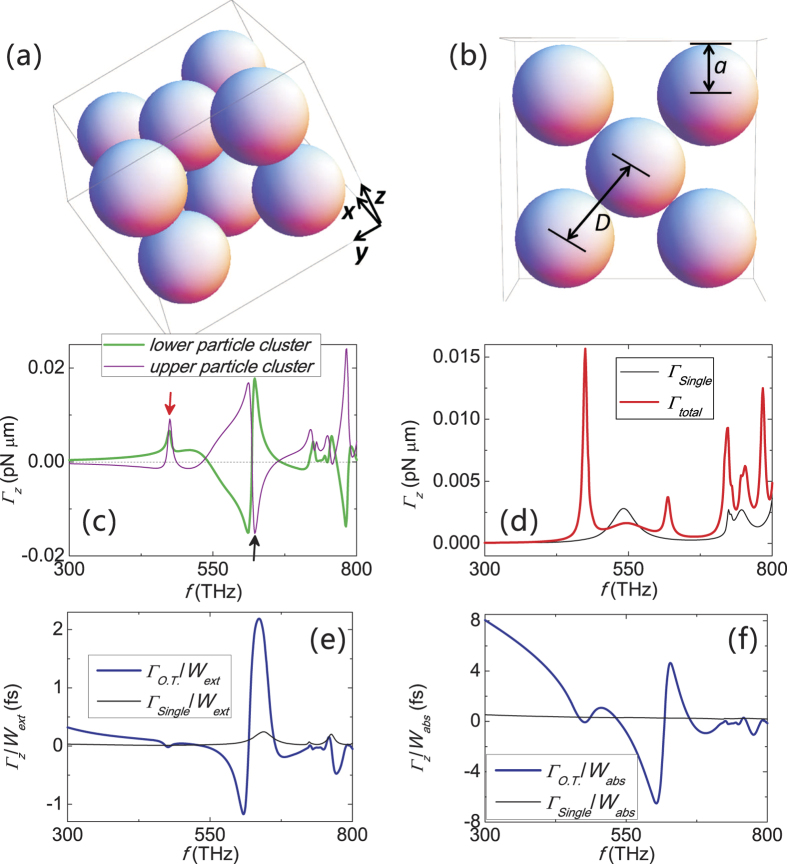
Optical torques acting on metallic structure, and the torque per unit light energy extinction and absorption rate. (**a**) A double-particle-cluster structure made of identical Ag spheres. (**b**) A single particle cluster made of identical Ag spheres. Radius = *a* = 30 nm. Separation between adjacent spheres is *D* = 60.8 nm. The optical torques for the upper and lower particle clusters are shown in (**c**), and the total torque for the double-particle-cluster structure (Γ_*total*_ = Γ_1_ + Γ_2_) and the torque for the single-particle cluster (Γ_*single*_) are shown in (**d**). We remark that Γ_*total*_ looks very different from the sum of Γ_1_ and Γ_2_, because Γ_1_ and Γ_2_ are almost equal and opposite, i.e. Γ_*total*_ is the residue of their sum after cancellation. Γ_*O.T*._/*W*_*ext*_ (Γ_*O.T*._/*W*_*abs*_) and Γ_*single*_/*W*_*ext*_ (Γ_single_/*W*_*abs*_) are shown in (**e**) and (**f**), respectively. The incident wave is a *z*-propagating plane wave with a modest intensity of 1 *mW*/*μm*^2^ and positive spin angular momentum.

**Figure 3:**
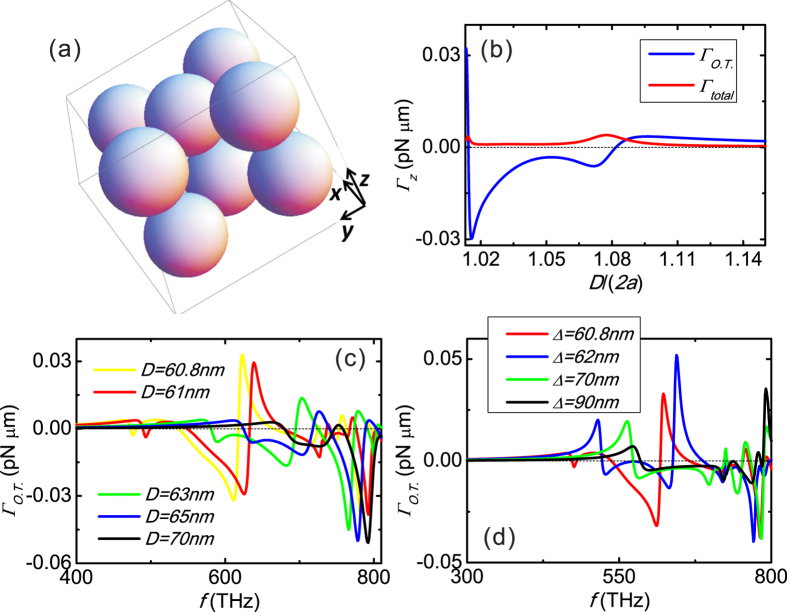
The role of gap to torque. The structure is shown in (**a**) with radius *a* = 30 nm. Optical twist and total torque of the double-particle clusters versus separation between adjacent spheres (denoted by “*D*”) are plotted in (**b**) with incident wavelength fixed at 482 nm (f = 623 THz). Optical twist versus frequency of incident light is also shown in (**c**) for different *D*. In (**d**), optical twist are shown by fixing the gap between the two particle clusters at 0.8 nm, while changing the intra-cluster gap (denoted by “Δ”) among the same layer. The incident wave is a *z*-propagating plane wave with a modest intensity of 1 *mW*/*μm*^2^ and positive spin angular momentum.

**Figure 4:**
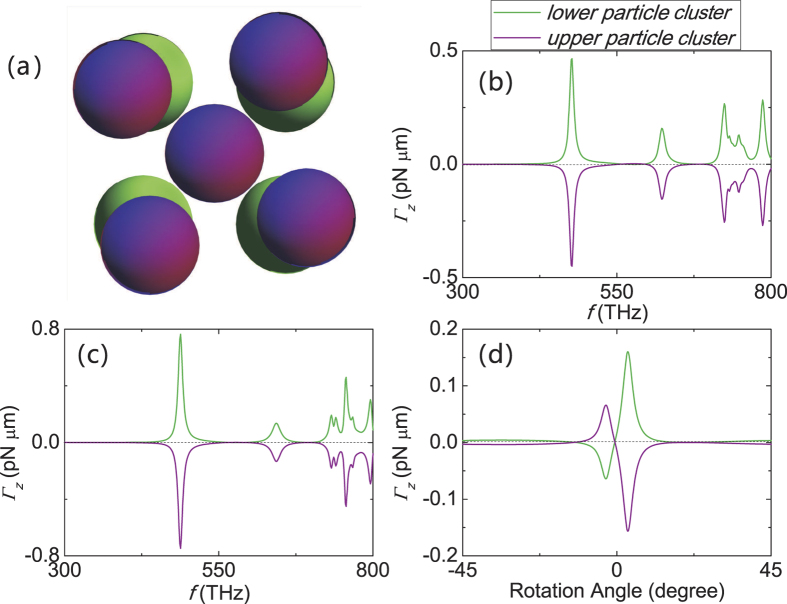
Optical torque for twisted double-particle-cluster structures. (**a**) The structure (made of Ag) is obtained by rotating the upper particle cluster (purple in color) of the double-particle-cluster structure used in Fig. 2(a), with *a* = 30 *nm* and *D* = 60.8 *nm*. Optical twist are plotted for this structure with the rotation angle equals to 3 degrees and 8 degrees in (**b**) and (**c**), respectively. (**d**) Optical twist versus rotation angle with wave frequency fixed at 623 THz. The incident wave is a *z*-propagating plane wave with a modest intensity of 1 *mW*/*μm*^2^ and positive spin angular momentum.

**Figure 5:**
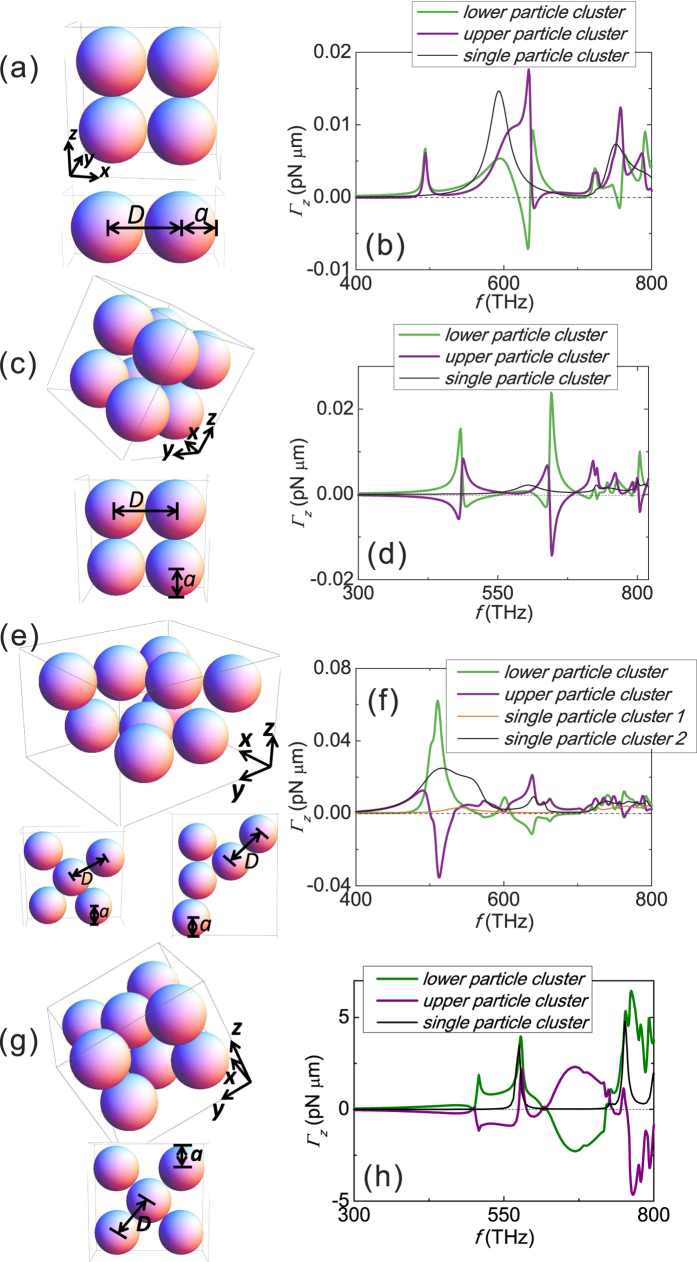
Optical torque for various geometries and beam profiles. The optical torques acting on structures (made of Ag spheres, *a* = 30 *nm* and *D* = 60.8 *nm*.) in (**a**), (**c**), (**e**) and (**g**) are plotted in (**b**), (**d**), (**f**) and (**h**), respectively. The torques of both single- and double-particle-cluster configurations are plotted. The incident wave is a *z*-propagating plane wave with a modest intensity of 1 *mW*/*μm*^2^ and positive spin angular momentum for (**a**–**f**), and the incident light is a z-propagating and x-polarized first-order Laguerre Gaussian beam with beam power 0.1 W for (**g**,**h**).

**Figure 6:**
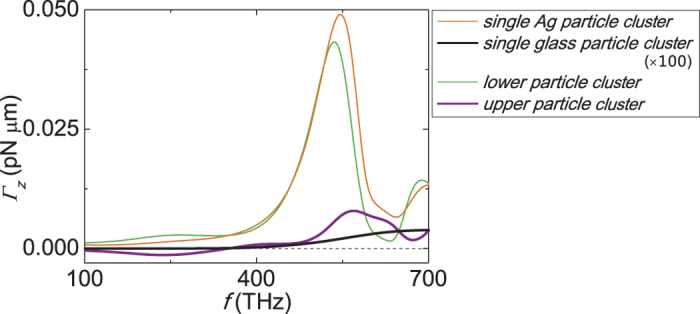
Torque on a dielectric structure enhanced by a nearby Ag structure. The double-particle-cluster structure is the same as Fig. 5(c) except *a* = 100 *nm* and *D* = 202 *nm*. Along *z*-direction, the lower particle cluster is made of Ag and the upper particle cluster is made of dielectrics (*ε*_*r*_ = 1.7689). Torque acting on the silver particle cluster and the dielectric particle cluster are denoted by the thin green line and the thick purple line, respectively. The torque for the single particle cluster of dielectrics (thick black line, the magnitude of torque is multiplied by 100) and Ag (thin orange line) are plotted for comparison. The incident wave is a *z*-propagating plane wave with a modest intensity of 1 *mW*/*μm*^2^ and positive spin angular momentum.

**Figure 7:**
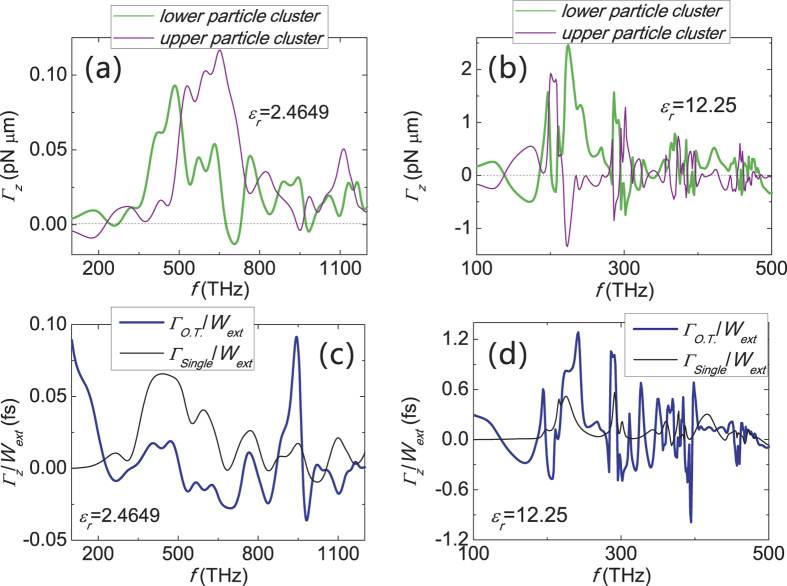
Optical torques acting on dielectric structures, and torque per unit light energy extinction and absorption rate. The double-particle-cluster structure is the same as Fig. 2(a) except *a* = 200 *nm* and *D* = 400.8 *nm*. (**a**,**c**) are for dielectric structure with *ε*_*r*_ = 2.4649. (**b**) and (**d**) are for high dielectric structure with *ε*_*r*_ = 12.25. The incident wave is a *z*-propagating plane wave with a modest intensity of 1 *mW*/*μm*^2^ and positive spin angular momentum.

We consider the optical torques acting on double-particle-cluster structures depicted in Fig. 1. To ensure the convergence of our calculations, each particle cluster of the structure is composed of a collection of identical metallic or dielectric spheres sitting on the same plane. Such configuration can be calculated accurately by using the generalized Mie theory for multi-spheres^38^,^39^. The incident light is a plane wave propagating along *z*-direction with positive spin angular momentum and an intensity of 1 *mW*/*μm*^2^. The origin of the torques acting on each layer are fixed at the center of mass of each particle cluster. The time averaged optical torque acting on the lower and upper particle clusters are computed by^12^


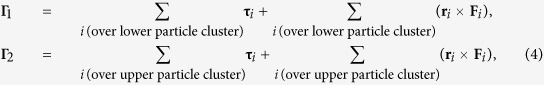


where


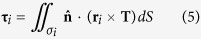


is the torque acting on the *i*-th sphere about its own center (vanishes for non-absorptive sphere), *σ*_*i*_ is the surface of the *i*-th sphere, **T** is the time averaged Maxwell stress tensor, **r**_*i*_ is the position vector of the *i*-th sphere measured from the center of mass of each particle, and


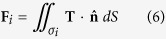


is the time averaged optical force that acts on the *i*-th sphere. The Maxwell stress tensor is evaluated by the generalized Lorentz-Mie scattering theory^40^,^41^,^42^,^43^,^44^,^45^. In short, the field quantities are expanded in a series of vector spherical wavefunctions. Then the expansion coefficients are obtained by applying the standard electromagnetic boundary conditions over the surfaces of every spheres with the help from the vector translation-addition theorem. This series expansion was truncated at some angular momentum *L*_*max*_ (=55 in this paper), which was chosen so that further increase in *L*_*max*_ does not change the results significantly. For a given particle distribution and dielectric constant, our approach subjects only to numerical truncation errors, but is otherwise exact within classical electrodynamics.

Alternatively, the time averaged optical torque can also be computed by


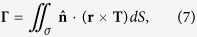


where *σ* is a surface enclosing the entire structure and **r** is the position vector measured from the center of the structures. Most of our results are computed from (4), and we have checked some of them against (7), with excellent agreements being achieved.

## Additional Information

**How to cite this article**: Chen, J. *et al*. Optical Twist Induced by Plasmonic Resonance. *Sci. Rep.* **6**, 27927; doi: 10.1038/srep27927 (2016).
